# Maternal and newborn healthcare utilization in Kampala urban slums: perspectives of women, their spouses, and healthcare providers

**DOI:** 10.1186/s12884-023-05643-0

**Published:** 2023-05-05

**Authors:** Andrew Magunda, Sam Ononge, Dorothy Balaba, Peter Waiswa, Daniel Okello, Henry Kaula, Brett Keller, Erica Felker-Kantor, Yvonne Mugerwa, Cudjoe Bennett

**Affiliations:** 1 Population Services International Uganda, UAP Nakawa Business Park Plot 3-5, New Port Bell Road, PO Box 8082, Kampala, Uganda; 2 Makerere University College of Health Sciences, PO Box 7072, Kampala, Uganda; 3 Kampala Capital City Authority, City Hall, Plot 1-3 Apollo Kaggwa, PO Box 7010, Kampala, Uganda; 4 Tulane School of Public Health and Tropical Medicine, New Orleans, USA; 5 USAID, Bureau for Global Health, Office of Maternal and Child Health and Nutrition, Research and Policy Division, Social Solutions International, 500 D, SW Cubicle 05.04.3P, Washington, USA

**Keywords:** Uganda, Maternal and newborn health, Urban slums, Qualitative research

## Abstract

**Background:**

It is assumed that the health conditions of urban women are superior to their rural counterparts. However, evidence from Asia and Africa, show that poor urban women and their families have worse access to antenatal care and facility childbirth compared to the rural women. The maternal, newborn, and child mortality rates as high as or higher than those in rural areas. In Uganda, maternal and newborn health data reflect similar trend. The aim of the study was to understand factors that influence use of maternal and newborn healthcare in two urban slums of Kampala, Uganda.

**Methods:**

A qualitative study was conducted in urban slums of Kampala, Uganda and conducted 60 in-depth interviews with women who had given birth in the 12 months prior to data collection and traditional birth attendants, 23 key informant interviews with healthcare providers, coordinator of emergency ambulances/emergency medical technicians and the Kampala Capital City Authority health team, and 15 focus group discussions with partners of women who gave birth 12 months prior to data collection and community leaders. Data were thematically coded and analyzed using NVivo version 10 software.

**Results:**

The main determinants that influenced access to and use of maternal and newborn health care in the slum communities included knowledge about when to seek care, decision-making power, financial ability, prior experience with the healthcare system, and the quality of care provided. Private facilities were perceived to be of higher quality, however women primarily sought care at public health facilities due to financial constraints. Reports of disrespectful treatment, neglect, and financial bribes by providers were common and linked to negative childbirth experiences. The lack of adequate infrastructure and basic medical equipment and medicine impacted patient experiences and provider ability to deliver quality care.

**Conclusions:**

Despite availability of healthcare, urban women and their families are burdened by the financial costs of health care. Disrespectful and abusive treatment at hands of healthcare providers is common translating to negative healthcare experiences for women. There is a need to invest in quality of care through financial assistance programs, infrastructure improvements, and higher standards of provider accountability are needed.

## Background

Despite remarkable achievements in reducing maternal and neonatal deaths, global maternal and neonatal mortality rates remain unacceptably high. In 2017, an estimated 295,000 women died due to pregnancy-related complications and childbirth [1]. Over 94% of these deaths occurred in low-resource settings with sub-Saharan Africa (SSA) accounting for approximately two-thirds of all maternal deaths [1]. Although global neonatal mortality rates have dramatically fallen since the 1990s, an estimated 2.4 million newborns died in 2019 in the one-month period following birth with the highest rates in SSA at 27 deaths per 1,000 live births [2].

Similar to trends across SSA, Uganda’s maternal mortality ratio (MMR) and neonatal mortality rate are high [1, 3]. Although rates of maternal mortality have decreased by 13% since 2011, as of 2017, the national MMR was estimated at 336 maternal deaths per 100,000 live births and the neonatal mortality rate was 27 neonatal deaths per 1000 live births [4]. While data from the 2016 Demographic Health Survey (DHS) show that the proportion of women receiving antenatal care (ANC) from a skilled provider increased from 95% in 2011 to 97% in 2016 and institutional childbirth increased from 57% in 2011 to 73% in 2016, utilization and quality of healthcare is highly variable and heterogenous across the country [5, 6].

Over the past few decades, development, and economic growth of urban centers in low-to middle-income countries (LMIC) has not kept pace with the large-scale migration from rural to urban areas, making it difficult for governments to provide affordable housing, basic social services, and employment. This has resulted in an unprecedented growth of slums and informal settlements on the periphery of urban cities [7, 8]. It is a commonly held assumption that the health conditions of urban populations are superior to their rural counterparts due to greater healthcare availability and economic opportunities [9, 10]. Evidence from urban poor communities across Asia and Africa, however, show that poor and marginalized urban populations have worse access to ANC and facility childbirth compared to the non-poor, and often have maternal, newborn, and child mortality rates as high as or higher than those in rural areas [7, 10–14]. In Kampala slums, for example, the estimated stillbirth rate is 43 deaths per 1000 live births which is more than double the 19 deaths per 1000 live births in rural areas of the country. This finding highlights the growing urban health disparities in the country and suggests that despite availability of healthcare, urban poor women in Uganda may not be accessing or receiving quality healthcare.

Understanding the factors that facilitate or impede women from utilizing healthcare is the first step to eliminating urban health inequities for mothers and newborns. Drawing on the perceptions and experiences of women, their spouses, and health providers, the aim of this study was to better understand the determinants of maternal and newborn healthcare utilization in two urban slum communities of Kampala, Uganda. Study findings will inform a new initiative to improve maternal and newborn health (MNH) in Kampala slums. This is important considering that more than half of Kampala residents currently live in informal settlements, and by 2035 the city will be home to more than 30% of Uganda’s population [15, 16].

## Methods

### Setting

**Fig. 1:**
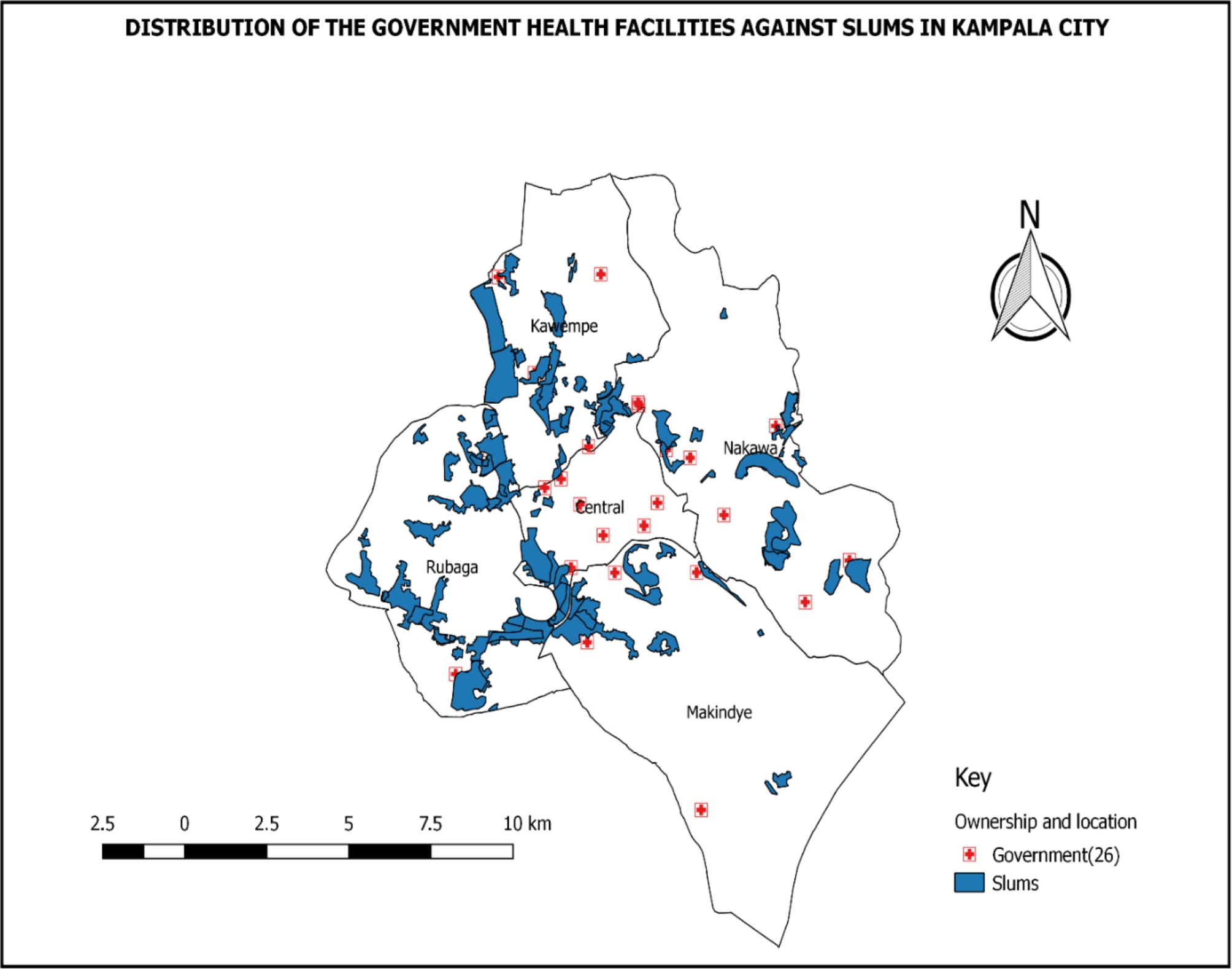
Map of Kampala city showing the informal settlements and facilities

The population of Kampala is estimated at 1.6 million and it is reported to be among the fastest-growing cities in SSA with an annual growth rate of 4.03% [15]. The city is divided into five administrative divisions: Kampala Central, Kawempe, Nakawa, Makindye, and Rubaga. These administrative divisions are home to 57 informal settlements or slums. As shown in Fig. 1, the MaNe project was implemented in Makindye and Rubaga divisions, which were purposively selected based on population density and the presence of informal settlements or slum communities. The informal settlements are overcrowded and lack basic infrastructure including waste disposal, sewage, electricity, and roads. Most slum residents live in small wood or brick houses with tin roofs and communal latrines and bathing areas [17].

The public health system in Kampala is managed by Kampala Capital City Authority (KCCA). Government and KCCA policy is that all residents receiving healthcare from public health system are exempted from any costs. However, few clients are faced with out-of-pocket expenses. Healthcare provision is through a mix of public and private facilities. In addition to providing healthcare, KCCA is responsible for the management and oversight of both public and private facilities, including the development and implementation of standard care protocols and ensuring that all providers are licensed and maintain their credentials. In the slum communities, maternal and newborn healthcare is available at formal health facilities and informally through community-based care provided by traditional birth attendants (TBAs). A 2017 health facility audit reported a total of 444 health facilities (5 public and 439 private) in Makindye and Rubaga, two divisions in Kampala that have the largest slum communities. Makindye and Rubaga divisions host 60% of all informal settlements in the city. Makindye division has 15 informal settlements, while Rubaga division has 13. Similar to other low resource settings, the poor women from slums often bypass seeking care at private and public primary level facilities for higher level referral facilities [18, 19]. As a result, referral facilities are overcrowded. To reduce congestion, KCCA improved infrastructure by renovating some public facilities and constructing additional wards for the patients.

### Study design

This qualitative study included in-depth interviews (IDIs), key informant interviews (KIIs) and focus group discussions (FGDs). Data were collected in two of the five divisions of Kampala. The two divisions, Makindye and Rubaga, were purposively selected because they are the most populous (> 50% of the population) and are home to the largest informal settlements in Kampala. Study participants were purposively selected. Table 1 shows the distribution of study participants by data collection type.

**Table 1:** Characteristics of study participants

Participant category	Number	Type of data collection	
Women who had given birth within 12 months	55	IDI	
Community leaders	3	FGDs	
KCCA and health providers	21	KII	
Coordinators of private ambulances and emergency medical technicians	2	KII	
Traditional birth attendants	5	IDI	
Husbands	12	FGDs	

The study population was at two different levels, the community and division levels. At community level the study participants included women who gave birth within 12 months prior to data collection, these should have delivered while residing in the slums and were residents at the time of data collection. Spouses/partners of the said women were engaged to seek their opinions on maternal and neonatal health issues. The other group at this level was the community leaders who included local council members, village health team members and opinion leaders. During the data collection, informal providers (traditional birth attendants) were included because they were pointed out as alternatives for the health facilities in health care provision for those that cannot afford the facilities

At division level, health care providers from private and public facilities that were selected purposively ensuring that those serving these populations were reached. These included both frontline health workers such as medical officers, midwives and nurses but also the health managers and proprietors of private facilities respectively and those involved in referral transport systems. In addition, the input of the KCCA health teams was sought both at city and division levels. The proprietors of private facilities were interviewed to profile private facilities which are the major providers of care for Kampala residents.

### Participant’s selection

At community level, women and spouses/partners of women, were identified by village health teams (VHTs) involved in MNH work. At division level, the health providers- facilities that serve the study population were identified using the HMIS data and DHIS data and KCCA reports. Providers from high serving facilities were targeted and identified through KCCA records but also snow balling supplemented by information from community members. A formal request was made to respondents for them to participate in the interviews. The interviews were conducted in the participant’s home, workplace, or facility and lasted for 45 to 60 minutes on average. FGDs and interviews were conducted by research team from Makerere University School of Public Health experienced in qualitative research. They were fluent in English, and Luganda (the local dialect language). Before each interview, the researchers clarified that they had no involvement in district administration, that the participant’s particulars would remain confidential, that their participation was voluntary and that responses would not lead to any retribution.

### Data collection

This study used an inductive approach to data collection. This approach allowed for capturing experiences and perceptions about maternal and newborn health within the broader context of the social and environmental setting of the slum communities. A semi-structured field guide with a series of open-ended questions was used to direct the interviews and FGDs with participants. The field guide included questions on maternal and newborn health knowledge, beliefs, and practices, experiences seeking and receiving maternal and newborn care, the role of men in healthcare decision-making, patient-provider relations, differences in public and private care, formal vs. informal healthcare, and quality of healthcare. All interviews and FGDs were conducted in Luganda, audio recorded and lasted approximately 60 minutes. Recordings and field notes were later translated and transcribed in English. Data collection occurred between February and April 2019.

Participation in the study was voluntary. Written informed consent was obtained from all study participants. The study received ethical approval from the Institutional Review Boards (IRB) of The AIDS Support Organization (TASOREC/003/19-UG-REC-009) and Uganda National Council for Science and Technology (HS327ES).

### Data analysis

Data were analyzed following a thematic analysis approach. Six members of the study team worked collaboratively on data analysis. First, a codebook was developed using codes based on the study objectives and common themes from the literature. Second, transcribed interview and FGD texts were imported into NVivo qualitative analysis software. One interview was selected and coded by each team member. The coded interview was compared to ensure consistency in application of codes. Third, the team conducted line-by-line coding of texts [20]. The process was iterative and when necessary, codes were added, removed, and amended based on emergent findings. The coded data were then organized into overarching themes. The present analysis focuses on the main themes extracted from the text and quotes from participants are included to contextualize study findings. The six main themes included: 1) Knowledge about MNH, 2) MNH care practices, 3) Decision-making on where to seek care, 4) MNH care provision, 5) Quality of care, and 6) Referral processes.

## Results

### Theme 1: Knowledge about maternal and newborn health

Knowledge about MNH was assessed by inquiring what participants, specifically women and their spouses or partners, knew about maternal and newborn illnesses, available healthcare, and best practices. Besides knowing pregnancy danger (e.g., vomiting, dizziness, fainting, bleeding, headaches, etc.) and common newborn health illness (e.g., flu, cough, fever, skin rashes, difficulty in breathing, etc.), most participants were unable to discuss when to seek care. Mothers considered febrile illnesses among newborns to be more severe ailments than the non-febrile illnesses. The mothers were less confident in describing either maternal or newborn illnesses. However, mothers or their newborns who had experienced the illness were able to recall the situation.

Participants’ knowledge of maternal healthcare was limited to ANC and childbirth with limited knowledge about postnatal care (PNC). Few women were aware of the benefits of PNC or available PNC services. When asked about PNC, women described the care they received after childbirth before discharge. They understood PNC as pre-discharge care and counselling before leaving the facility. Participants did not mention attending PNC visits and only returned to the health facility for scheduled immunizations or emergencies. In addition, healthcare workers perceived women to have limited knowledge about MNH which they correlated to poor healthcare-seeking behaviors and practices. Several healthcare providers explained that women from slum communities tended to seek care late and had little knowledge about the types of healthcare available for pregnant women or interventions that may be needed for high-risk cases. They noted that many of the women who seek care late in pregnancy refuse the recommended healthcare and resort to using more traditional practices such as the use of herbal medicine. One provider explained,

The challenge we face is that most of these women they are not aware, they come in when most of them didn’t go for antenatal, they do not know the importance of going to a health center for ANC. [...] at delivery and they come at the last minute... she is a prime gravida (first time pregnancy), she doesn’t know that this height was going to be operated [...] some of them they do not do scans, you find that even if the baby is in the wrong position but for her, she is saying that I have to push.

[*KII, health provider, public facility*, Rubaga division]

In general, most women understood the importance of seeking ANC from a formal health facility during pregnancy. Women perceived ANC as a personal health benefit, stating that ANC visits improved their knowledge on self-healthcare practices during pregnancy, in particular eating healthy, avoiding toxic foods, and rest. They also viewed ANC as an important determinant of having a healthy baby. Despite reporting that ANC was a critical factor for preventing complications during pregnancy, most women did not know when to seek care. Most women stated that ANC visits should start at five months into pregnancy. Differences in knowledge about ANC were noted between first-time mothers and women who had previously delivered at a health facility. Older women were more aware of the benefits of seeking ANC early. According to one participant,

Now like I always get stomach pains during pregnancy and so I start attending ANC as early as one month because I don’t want to stay at home and lose my baby when there are people who can help me professionally. Even going for scan, I can do it twice or thrice just to know that the baby I am carrying has no complications.

[IDI, older mother, Makindye division]

Participants largely understood the benefits of delivering at a health facility and expressed a preference for facility-based births. The most common reasons women decided to deliver at a facility rather than at home were the access to skilled providers and equipment which increased the chances of having an uncomplicated and healthy birth.

Knowledge about MNH was obtained through prior experience seeking MNH services at facilities and through social media, friends and family, TBAs, and myths and cultural traditions. First-time mothers and adolescents relied on social media, social networks, or traditional customs for information. As one adolescent mother explained,

*“In most cases, I always get that information from television. There are those programs I usually watch about health issues, like there is a program about health that I usually watch on Bukedde Television. They always tell us the foods that we should eat as pregnant mothers, and there is another program on Spark Television on weekends also about health, they are very interesting.” [IDI, adolescent mother, Rubaga division]*

#### Male knowledge of maternal and newborn health

Findings from the FGDs with spouses and partners of women who had recently delivered revealed that men had minimal knowledge about MNH. Most men were oblivious to women’s needs during pregnancy and viewed pregnancy and parenthood as a female domain. Several adolescents and young men expressed frustration at not being able to support their partners during pregnancy due to lack of knowledge about pregnancy and childbirth. To improve male involvement in the pregnancy process, participants recommended an increased focus on male sensitization of MNH at the community and facility-level.

“*The sensitization is also needed. They should invite us to the health facility and sensitize us about what we should do to support women during childbirth and other preparatory activities.” [FGD, adolescent spouse/partner, Makindye division]*

### Theme 2: Maternal and newborn healthcare-seeking practices

Maternal and newborn healthcare-seeking practices were heavily tied to knowledge about MNH. Women who had previous experiences with pregnancy and birth were more likely to employ recommended healthcare-seeking practices. Adolescents and first-time mothers relied on advice from family members and social networks on what behaviors to practice. Birth preparedness, attending ANC visits, and seeking care for pregnancy-related illness were the most frequently practiced behaviors.

#### Birth preparedness

Birth preparedness was described as acquiring the essential materials needed for childbirth such as baby clothes, mackintosh sheets, mama kits, and basins for washing. Arranging for a caretaker during childbirth was also part of the birth preparedness planning process. Mothers and sisters were the primary caretakers during childbirth. They also helped look after younger children while the woman was in the health facility. Participants also mentioned planning for hospital bills and transportation costs. Many women and their spouses attempted to save money prior to childbirth to finance private transportation and unforeseen medical costs. A few participants who had reliable income were able to start saving from the beginning of pregnancy. A pregnant mother stated,

As for me now, like I do a lot of things now like every week, I make sure that I buy some things for the baby and other things to use like gloves and mama kit. I also have a small box where I save money. I make sure I save money, I put it in that box every day so that when the time reaches for me to go to the hospital to give birth, I have some money and I can be able to take care of myself in case there is anything which needs money.

[IDI, pregnant woman, Rubaga division]

#### Antenatal care

Most women sought ANC at public facilities due to the higher cost of care at private clinics. Women generally attended their first ANC visit late with some starting as late as 7 months into pregnancy. Reasons for late attendance included lack of knowledge about when to start seeking care and lack of money. Women who did seek ANC early were usually those who had a history of pregnancy complications. Women noted that health workers encouraged them to attend ANC visits with their spouses or partners, but they received little male support. Women understood low male involvement to be associated with men’s fear to test for HIV and belief that pregnancy was a female responsibility. According to men, lack of male involvement was due to lack of time and their financial responsibilities.

#### Care-seeking behavior for illness

For treatment of maternal illnesses, mothers sought care from formal and informal providers. The practice of seeking formal care for pregnancy-related illnesses was heavily influenced by healthcare practices of family and friends and financial means. When women were unable to seek formal care due to financial restrictions, they often turned to informal medicine such as the use of herbs for nausea and fatigue. For newborn illnesses, women reported seeking care for the conditions they considered serious such as febrile illnesses. In most cases, they went back to the health facility where the baby was born. Illnesses perceived as minor such as the common cold and flu, were treated with over-the-counter medicines, traditional medicine, and household remedies. An adolescent mother described the process of treating her baby’s illness,

*“…Yes, he suffered from skin sores, and they were big and when you squeeze them, they would bring out puss… I was told to use local herbs. I bathed him in tomatoes, because they said the disease was called “ebinyanya”, and so they said it was tomatoes that could heal him. So, I used to bathe him with tomato leaves… Yes, they helped, he healed and remained with scars.” [IDI, adolescent mother, Makindye division]*

### Theme 3: Decision-making on where to seek care

Over the course of pregnancy, women and their families made decisions about where to seek ANC and childbirth care. The decisions were influenced by multiple factors including prior experience with facilities and health providers, accessibility of care based on proximity and cost, and intra-household dynamics between family members.

#### Household dynamics

While decision making on where to seek care varied across households and family dynamics, in most cases, it was either the woman or the man who decided where healthcare should be sought, but rarely were decisions made jointly. The choice of health facility usually depended on household finances which the spouse or male partner managed. Some women stated their desire to attend a private facility, but due to limited access to household finances they had to attend a public facility. In other instances, women were handed an allotted amount of money for healthcare expenditures, and it was up to them to decide how it was spent. Women who lived alone had more freedom to choose where to seek care, but finances were limited given they were the sole earner in the household. For adolescent girls, decisions were primarily made by their parents or in-laws because most of them were dependents and lived with adults.

*“No I don’t make any decision on my own, it’s my mother in law who is in charge of my life, because when I got pregnant, my father took me to my husband’s parents’ house and told my mother in law that she is now in charge of my life, he even sent me away from home, so now she is the one who controls me, that’s why she makes every decision for me, because she is the one who takes care of us, for me I do not have money, she is the one who provides everything.”* [*IDI, adolescent mother, Makindye division]*

#### Affordability and accessibility

Financial security and affordability were the primary determinants influencing where women sought care. Most women would preferred to attend private facilities as they perceived the care to be higher quality. However, financial insecurity prevented them from seeking care at private facilities. Most women sought care at public facilities because the healthcare were supposed to be free or cheaper than private facilities. A few participants had prior experiences delivering at private facilities and they reported switching to public facilities for subsequent births due to the high cost of private healthcare.

*“Money was the only reason as to why I considered choosing to go to Nsambya barracks [public health facility], all services there are free, yet at private [facility] when I asked, they told me a lot of money so I could not manage, that’s basically the only reason as to why I went there [Nsambya barracks].” [IDI, older mother, Makindye division]*

Accessibility of healthcare was also an important factor in deciding where to seek care. Participants indicated a preference for facilities closest to home as it saved cost in transport and time. Closer facilities were also viewed as preferable in case of obstetric emergencys or early labor.

#### Prior experience

In addition to affordability and accessibility, prior experience with a facility or health provider played a significant role in deciding where to seek care. Women discussed wanting to attend care where health providers were skilled, respectful, and could assure them of a safe and healthy childbirth. Women who had a previous positive experience with a particular provider generally opted to return to the same facility if finances allowed. Family and friends’ experiences with providers and health facilities were also considered when choosing a location for care.

*“Because I could see she was able to deliver me…Because she is knowledgeable about things to do with delivering women and she had delivered many women in this area, so I also decided to go there.” [Adolescent mother, IDI, Makindye division]*

*“… I heard some women talking and I heard them commenting that Naguru is a good health facility. So when you speak and someone says that if I also become pregnant, I will go there.” [IDI, older mother, Makindye division]*

### Theme 4: Maternal and newborn healthcare service provision

According to study participants, formal and informal care were available in the slums. Formal care settings included public, private for-profit (PFP) and private not for-profit (PNFP) health facilities. Preventive, diagnostic, and curative healthcare were provided by formal facilities. Informal care was identified as home-based care provided by TBAs. Formal maternal care was described by providers as ‘standard care’, including health education that focused on birth preparedness, the importance of attending all ANC visits, routine micro-nutrient supplementation of folic acid and iron, and HIV and sexually transmitted disease screening. Informal care provided by TBAs were typically home-based and consisted of herb treatment for pregnancy-related illnesses. Some of the herbs are aimed at enlarging the vagina and pelvic bones of the woman in preparation for childbirth, usually given as a herbal drink, applied to private parts or sitz baths. They also provided counselling and cleaning of caesarean section wounds.

Interviews with formal providers revealed a stark division between public and private health facilities with little collaboration. Some TBAs reported having collaborations with formal facilities where they referred women for scans, immunizations, diagnostic testing, and complications during childbirth. However, it was also noted that some formal providers expressed anger at women and TBAs when a patient came in during labor due to complications. One TBA mentioned that the formal and informal divide was an issue when it came to reporting of maternal deaths, such that home-based births were often not officially registered.

### Theme 5: Quality of care

The poor quality of care provided at public health facilities was a major concern among women and acted as a deterrent for seeking formal MNH services. The central concern about quality was the poor treatment as well as the common practice of facilities requiring bribes or informal payments to secure treatment. From the perspective of healthcare providers, the quality of care rendered were dependent on facility infrastructure, access to essential medicines and equipment, patient-provider ratio, and individual provider motivation.

#### Disrespectful care

Women who sought care at public facilities expressed dissatisfaction with the quality and interpersonal care, specifically the disrespectful and abusive treatment by health workers. Women reported being yelled at, ignored, and mocked for being pregnant. They described health workers as negligent, uncaring, abusive, and generally having a bad attitude. Many women described scenarios where women were left alone during labor and feared asking for help. Women recalled instances where women were refused care or left unattended because they didn’t have the “mandatory” birth items such as gloves or mackintosh sheets. For example, one women explained,

*“It’s a government hospital but the health workers some of them are very rude. Now like when I was there, there is this woman, who was there, the health workers were not caring about her. I think by mistake her water broke when she was there; immediately the health worker who was on duty came, instead of taking her to labor suit, she pulled her hair and shouted at her, telling her that “you’re not supposed to deliver from there”, abused her, yet she was the one not taking care of her. It’s even us who called her, some of those health workers have no mercy, they talk to us rudely yes.” [IDI, mother, Makindye division]*

Participants also complained that there was little consideration for patients’ welfare and property by staff in public facilities. For example, they reported that patient and family belongings were often disregarded by cleaning staff becoming soaked or ruined since they had nowhere to store items but on the floor. A spouse of a women who had recently delivered stated,

*“In such hospitals, many of the pregnant women, together with other patients are made to sleep on the floor and put their belongings on the floor. When the cleaners come to clean, they simply pour water without letting the people know. Their belongings end up being soaked in the water. You people who are doing this study need to visit such facilities to see for yourself.” [FGD, spouse, Makindye division]*

It is important to note that not all women had negative experiences with care at public facilities. A few women described positive experiences reporting that the health providers were caring, responsive, and attentive especially during labor. However, this acknowledgement was often viewed as an exception to the standard poor quality of treatment.

*“During that time of giving birth, like when you are experiencing labor pains and you start screaming that the baby is coming, she could come to you quickly, checks you, and sits beside you. Then the last time when I called out, she hurried, came and put-on gloves and delivered my baby. It is not like others who will start quarrelling that you called me earlier and yet you were not ready to deliver.” [IDI, older mother, Makindye division]*

When providers were asked about the quality of healthcare rendered to the slum communities, many became defensive and described the challenge of providing adequate care in resource poor conditions with a population of low health literacy. Many health workers expressed lack of motivation as they felt their work and commitment were not recognized or appreciated. Additionally, there was little incentive for providers to offer quality healthcare as they received minimal support or opportunities to advance their training. Lack of oversight along with provider frustration, dissatisfaction, and low motivation were likely related to the poor quality of healthcare.

#### Informal payments and bribes

Participants described the common practice of health workers seeking bribes or requesting informal payments before healthcare would be provided. It was not uncommon for health workers to threaten patients and refuse to provide care until they had been paid. Women cited instances where providers demanded payment after childbirth and would not discharge the new mother until paid. In some cases, women and their families had not prepared for this extra cost as they thought they were going to receive free care at a public facility. Participants perceived the poor treatment by providers to be associated with this system of informal payments. According to one woman from Rubaga division, “*in order to treat you well you have to pay them [health provider] some money…to pay attention to you…[so] I gave them 60,000 Uganda shillings.”*

This system of informal payments did not occur at private facilities which typically asked for formal payment after birth and patients could have the option of paying in installments. Furthermore, women attending private facilities had planned for the cost unlike women who went to public facilities who thought they would receive free care, but ultimately had to pay bribes to receive healthcare.

#### Stock-outs, equipment shortages, and poor infrastructure

Participants, including providers, described public facilities as overcrowded with inadequate space to serve the high volume of patients. Women explained that due to the inadequate space and limited beds they had to sleep on the floor with their babies or they had to share beds with other patients. They also perceived early discharge following birth because of limited space.

*“They told us to move to corridors and I slept down together with other women, on cement, no mattress, nothing, remember with the baby. There were no beds, all the beds were occupied. That made me uncomfortable, so, I laid there a bed sheet and I slept with my child.” [IDI, older mother, Makindye division]*

Providers at both private and public facilities also discussed lack of space as a major barrier to providing quality healthcare. Many of the private facilities were particularly small which made it difficult for providers to attend to multiple patients at once or to devote space specifically for maternal and newborn healthcare. These limitations were directly tied to worse quality care, frustrated providers, and an increase in referrals, which created an extra financial cost for the patient.

*The women do not have a place for resting before going into labor. There is no walk area for the women in labor. I do not have a wheel chair to assist in moving tired mothers so we have to carry them. Sometimes we have no power and we have to use a torch. We also have no running water, I use jerry-cans for carrying water* (**PFP, Lungujja**)

Shortages and stock outs of essential medicines were another barrier that influenced the quality of care provided to patients. Providers at public facilities stated that shortages of basic supplies such as gloves, sterilizers, cord scissors, and fetal forceps were quite frequent and without the proper supplies they could not treat patients and often had to refer them to other facilities. Additionally, due to these shortages, women were expected to purchase the supplies themselves and arrive for childbirth with the proper supplies in hand including gloves, sheets, surgical suture, and medications. These additional costs prevented some women from obtaining care at a facility or from buying the recommended medication to treat illness. One health provider described his frustration with medicine stock outs and patient financial constraints,

The main challenge is drug stock-out, like for the ferrous Sulphate, Fansidar, the antibiotics which are needed for pregnancy, you find that they are out of stock. You find a mother with a urinary tract infection and you write for her, and she cannot buy because she has no money. She even tells you how she has no transport to get to the health center and how do you expect her to get better?

[*KII, health provider, public facility*, Makindye division]

Lack of essential medical equipment such as ultrasound scanners, incubators, and oxygen cylinders were a major source of stress and frustration for providers. One provider described that she was unable to provide treatment because she was missing the apparatus to operate the oxygen cylinder. As a result, she had to refer the patient to the emergency room for oxygen therapy.

Overall, the inadequate infrastructure and lack of resources at health facilities was perceived by women and providers as a barrier to accessing and receiving quality healthcare. The inability to provide basic and essential healthcare to their patients was tied to provider frustration, bad attitudes, and lack of empathy for patients. The poor treatment by providers, in turn, increased dissatisfaction among women and their families and greater hesitancy to seek formal care.

### Theme 6: Referral processes

The process of referring a patient was an emergent and reoccurring theme from KIIs with health providers. Providers discussed the common reasons for referral, the functionality of the referral process, and transportation for referrals.

#### Referral practices

The most common reason for referring a patient was lack of the health facility capacity. Such cases included those that required more advanced diagnostic tests and major surgeries, while others were due to shortages of supplies. The second most frequent cause of referral was complications and risk based on the judgment of the provider and the client history. Women who needed 24-hour attention were typically referred to hospitals because lower level facilities (health centre IIIs and IIs) majorly, functioned during day hours.

*We refer complicated [diseases] like malaria, we give them first aid and then refer because in most cases we do not work 24 hours so we cannot manage to monitor them throughout the night except when they come in at late hours and they do not have the means of going somewhere else. Then there are conditions of people who come when they have severe infections, for example, if we test a mother positive for HIV, we normally send them to hospitals to get more attention because we cannot handle them here*.

[*KII, health provider, public facility*, Makindye division]

#### Referral procedures

The interviews with health providers revealed that the health system had no standard protocol for referral. The referral process typically involved the patient receiving a note from the health provider. The referral note contained contact information of the referring facility and details of the patient’s condition. There were also no standard procedures for follow-up with referrals. In the rare instances that follow-up did occur, it was by telephone. Follow-up by phone, however, presented challenges in that many patients did not own their own mobile phone. As one provider who worked in a public facility in Makindye division explained, *“…that’s the biggest gap we have. I normally try to reach them on phone and those who do not have phones unfortunately it becomes difficult for me to follow them up.”*

#### Referral transportation

Providers commented on the lack of transport options for transferring a patient from one facility to another. Access to ambulances was described as limited and even when ambulances were available, they were often not equipped or were too expensive for the patient. In most cases, women were required to make their own transportation arrangements, and due to cost, ended up using public transport..

That is a difficult process because we have no vehicle or a motorcycle. We get a boda-boda like those of Safe boda (motorcycle) and tell him to take the patient to Kawempe or Mulago hospital. And imagine she is in a lot of pain. So, you escort her to the hospital and our roads are very bad...it is a very big problem for us.

[*KII, health provider, private facility*, Rubaga division]

## Discussion

Findings from this study indicate that women sought ANC and delivered at public health facilities, despite the relatively small number of public facilities. This results in congested public facilities and low quality of care. To decongest the public health facilities, KCCA undertook renovation and increasing the bed capacity of the few public facilities. This resulted in more mothers being attracted to the facility and did not result in anticipated decongestion. The KCCA administration was designing strategies to source out private facilities to offer MNH care. Although women were generally knowledgeable about the benefits of ANC and facility-based childbirth, they typically sought ANC late and relied on TBAs for treatment of first trimester pregnancy symptoms. The use of herbs was mentioned as a common practice for treatment of pregnancy-associated illnesses. Women were less knowledgeable about PNC for themselves and their newborns and only mentioned seeking post-delivery care for scheduled immunizations.

The study findings provide insight to the decision-making process about where women seek care and the involvement of men in pregnancy and childbirth. Similar to other research, decision-making on where to deliver was rarely a joint process between women and their spouses or partners [21, 22]. Decisions were made by the woman herself or by the spouse and were highly dependent on household finances. In households where men controlled the finances, women had little say on healthcare expenditures and had to make do with the finances they were allotted. Overall, male involvement in pregnancy and childbirth was minimal despite efforts by healthcare providers and women to increase male participation. Men viewed pregnancy and childbirth as the woman’s domain and saw their primary responsibility as financial. Other factors that influenced decision-making on where to seek care included accessibility of healthcare, prior experiences, and recommendations by friends and family.

Women interviewed had negative childbirth experiences due to financial constraints and the quality of healthcare rendered. Most women perceived private facilities to have higher quality of care but were unable to seek care at those facilities due to the high cost. The quality of care provided at public facilities was described as disrespectful, discriminatory, and abusive. Women were often left alone to deliver without the help of a trained provider. Disrespectful care deterred some women from seeking care in the future. These findings are consistent with a systematic review which found that experiences of mistreatment during childbirth were prevalent in many countries across the globe [23, 24]. The interaction between health providers and patients is not just clinical; poor communication and low provider motivation can disincentivize patient care-seeking behaviour. Improvements in interpersonal communication and quality of care needs to be part of the health system strengthening process.

The study also highlights the large financial burden of the cost of childbirth has on families. Although public healthcare in Uganda is supposed to be free, women and their families reported having to provide essential resources such as surgical suture, mackintosh sheets, and birthing kits at the time of childbirth. The cost of these resources as well as transportation fees and informal payments to providers prevented some women from accessing formalized care. Women consistently reported that in many public facilities providers would not treat them unless they were paid an informal fee. The informal payments were made for treatment to initiate or to receive drugs that were meant to be free. The negative impact is of particular concern especially for the poor, because they bear the greatest burden of informal payments [25]. Several other studies have shown that inability to pay informal payments was associated with limit access to respectful care, delayed care-seeking, and forgone care [26–28]. In Tanzania, some healthcare providers feigned stockouts of drugs and supplies and sought of money from patients disguising to purchase the commodities from private market [29]. Other studies have reported similar findings that high out-of-pocket costs were one of the main barriers to women accessing healthcare [30, 31]. A study by Hossain et al., for example, found that 26% of women living in Dhaka’s urban slums reported high informal costs for why they did not use hospital for childbirth [32].

Interviews with healthcare providers revealed larger systemic problems with the Ugandan healthcare system. Health providers stated that there was minimal investment in the development of the health sector which often led to frustrated staff. Providers received little support or recognition from supervisors/facility managers and had to work in facilities that had minimal equipment, frequent medicine shortages and inadequate infrastructure. When infrastructure is particularly poor, it can result in further marginalization and exclusion of the poor. Studies have found that public and private facilities in poor urban areas that lack basic essential equipment in addition to lack of accountability can led to poor provider performance and low motivation, which has direct implications on patient care experiences [33, 34]. The unstandardized referral process, ambulance shortages, and minimal collaboration between private and public facilities as were other systemic problems that impacted the quality of care.

This study is not without limitations. Few women in the study had ever received care from the private sector. As such, findings may not be reflective of the perspectives and experiences of women who attended private facilities for maternal and newborn healthcare. Attempts were made to select women who had pregnancy and childbirth experiences at both public and private facilities. Study findings may be subject to social desirability bias as participants may have responded to reflect what they thought the interviewer wanted to hear. Eligibility for the study included women who had delivered in the 12 months prior to data collection. This may lead to recall bias as 12 months is a long time for women to recall experiences of care. Despite these limitations, the findings from this study have policy and programmatic implications for urban slum populations. Future programs should include a greater focus on health education for women and their spouses to improve knowledge about timeliness of care-seeking, how to access care, what types of MNH services are available, the importance of male involvement, and financial support opportunities. From a policy perspective, financial assistance for healthcare should be offered to households below a certain income threshold and women should be the primary beneficiaries. Examples of financial assistance programs include voucher schemes for free care at the point of service and conditional cash transfer programs that provide women with cash for family health purposes [35] .

Addressing the low standard of care provided to women in slum communities, including the disrespectful treatment by healthcare workers, should be prioritized. Providers should receive training on patient communication strategies and how to provide culturally appropriate care. Patients should be encouraged to voice their concerns and demand quality healthcare. The World Health Organization (WHO) provides strategies to address disrespectful care. The strategies include providing support through informed choice, access to foods and fluids, assuring women receive information to make informed decisions about care, access to a care companion, and confidentiality [36].

Finally, there is a need for substantial investment in facility infrastructure, improved coordination with the national supply chain to ensure supplies are allocated on time and to the facilities with greatest need, systematic referral procedures, collaboration between public and private facilities, and better regulation of health providers by KCCA. Providers should be held accountable for the quality of care they provide. At the same time, providers should be rewarded for their performance through formal recognition and opportunities to receive further training.

## Conclusion

Urban poor women living in slum communities of Kampala confront many challenges to accessing maternal and newborn healthcare. Despite availability of healthcare, many women and their families are burdened by the financial costs of care even at public facilities where care should be free. Not only do the out-of-pocket costs make care inaccessible for women, but also exclude them from high quality care as they are often expected to pay bribes to providers to receive treatment. Disrespectful and abusive treatment by health providers is common in slum communities translating to negative healthcare experiences for women.

## Data Availability

The datasets generated and analysed during the current study are not publicly available due non de-identification of the participants in the dataset, However, it will be available from https://data.usaid.gov/Maternal-and-Child-Health/MaNe_Uganda_Formative_2019_doc/x82b-mcbw after de-identification and on reasonable request.

## List of Abbreviations

ANC: Antenatal care
DHS: District health Services
FGD: Focus group discussion
HIV: Human Immune deficiency virus
IDI: In-depth interview
IRB: Institutional review board
KCCA: Kampala Capital City Authority
KII: Key informant interview
LMIC: Low to middle income countries
MNH: Maternal newborn health
PFP: Private for profit
PNC: Postnatal care
PNFP: Private not for profit
TASO: The aids support organization
TBA: Traditional birth attendant
